# Hyperbaric Oxygen Exposure Attenuates Circulating Stress Biomarkers: A Pilot Interventional Study

**DOI:** 10.3390/ijerph17217853

**Published:** 2020-10-27

**Authors:** Jae Seung Chang, Eunha Chang, Yoonsuk Lee, Yong Sung Cha, Seung-Kuy Cha, Won Gil Cho, Yangsik Jeong, Hyun Kim, Kyu-Sang Park

**Affiliations:** 1 Mitohormesis Research Center, Yonsei University Wonju College of Medicine, Wonju, Gangwon-Do 26426, Korea; godbless@yonsei.ac.kr (J.S.C.); eunhachang93@gmail.com (E.C.); skcha@yonsei.ac.kr (S.-K.C.); wch01@yonsei.ac.kr (W.G.C.); yjeong@yonsei.ac.kr (Y.J.); 2 Department of Physiology, Yonsei University Wonju College of Medicine, Wonju, Gangwon-Do 26426, Korea; 3 Department of Emergency Medicine, Yonsei University Wonju College of Medicine, Wonju, Gangwon-Do 26426, Korea; yslee524@gmail.com (Y.L.); emyscha@yonsei.ac.kr (Y.S.C.); 4 Department of Anatomy, Yonsei University Wonju College of Medicine, Wonju, Gangwon-Do 26426, Korea; 5 Department of Biochemistry, Yonsei University Wonju College of Medicine, Wonju, Gangwon-Do 26426, Korea

**Keywords:** hyperbaric oxygen therapy, biomarker, mitochondria, oxidative stress

## Abstract

Hyperbaric oxygen therapy (HBOT) has been used to provide oxygen to underperfused organs following ischemia or carbon monoxide intoxication. Various beneficial consequences of HBOT have been reported, including wound healing, anti-inflammatory action, and cell survival; however, the molecular mechanisms underlying these effects have not been elucidated yet. We applied a single HBOT program consisting of administration of 2.8 atmospheres absolute (ATA) for 45 min, followed by 2.0 ATA for 55 min, to 10 male volunteers without any metabolic disease. Within 1 week of HBOT, there was no alteration in serum biochemical variables, except for an increase in triglyceride content. As a mitochondrial stress indicator, the serum concentration of growth differentiation factor 15 was reduced by HBOT. The circulating level of γ–glutamyltransferase was also decreased by HBOT, suggesting an attenuation of oxidative stress. HBOT increased adiponectin and reduced leptin levels in the serum, leading to an elevated adiponectin/leptin ratio. This is the first study to investigate the effect of HBOT on serum levels of metabolic stress-related biomarkers. We suggest that HBOT attenuates mitochondrial and oxidative stresses, and relieves metabolic burdens, indicating its potential for use in therapeutic applications to metabolic diseases.

## 1. Introduction

Hyperbaric oxygen therapy (HBOT) is defined as an intervention in which the recipient breathes nearly 100% oxygen with a pressure of at least 1.4 atmospheres absolute (ATA). In clinical settings, the primary purpose of HBOT is to provide oxygen to insufficiently perfused lesions in the case of ischemic tissues or carbon monoxide intoxication [1]. However, secondary benefits have been reported from a high partial pressure of oxygen, including the induction of growth factors for accelerated wound healing and neovascularization [2,3]. HBOT also attenuates neuronal degeneration and apoptotic cell death after neurologic insults [4,5]. Furthermore, HBOT exhibits anti-inflammatory effects related to the suppression of oxidative stress and cytokine release [6,7,8]. Chronic exposure to hyperbaric oxygen has been shown to facilitate the recovery from injuries such as burns, bone fractures, and muscle injury [9,10].

Interestingly, HBOT affects the function of mitochondria, which serve as a major organelle for cells to use oxygen. HBOT was found to augment ATP synthesis in rat striated muscles and protected against pathologic deterioration of mitochondrial functions in motor neuron disease mouse models [11,12]. The loss of mitochondrial membrane potential caused by brain injury was recovered by HBOT [13]. Consistently, HBOT enhances endurance exercise performance by upregulating mitochondrial biogenesis and function [14].

It is well known that functional impairment of mitochondria by superoxide production participates in the development of metabolic and age-related morbidities [15]. Vicious cycles among oxidative stress with mitochondrial and endoplasmic reticulum (ER) stresses are critically involved in the pathogenic mechanisms of chronic metabolic diseases, including type 2 diabetes and metabolic syndrome [16]. To overcome these cellular stresses and abate the pathologic progression of metabolic diseases, the body elicits the integrated stress response (ISR) to enable metabolic and mitochondrial flexibilities. One aspect of the ISR is the secretion of mitochondrial stress-inducible humoral factors, fibroblast growth factor 21 (FGF21), and growth differentiation factor 15 (GDF15) [17]. However, sustained and uncompensated mitochondrial stresses maintain elevated levels of circulating FGF21 and GDF15; thus, the serum concentration of these factors indicates the presence of mitochondrial stress, which enables them to be used as biomarkers for hereditary mitochondrial diseases [18]. Recent reports have suggested that high levels of circulating FGF21 and GDF15 reflects the presence of mitochondrial stress and predicts chronic metabolic diseases [19,20].

In this study, we investigated the effect of HBOT on the metabolic and mitochondrial parameters of human volunteers, which has not been investigated before. After just one bout of HBOT, the levels of serum biomarkers reflecting mitochondrial and oxidative stresses were attenuated with improved adiponectin/leptin ratio, suggesting a relieved metabolic stress in response to HBOT.

## 2. Materials and Methods

### 2.1. Study Participants and Ethical Approval

Human volunteers were middle aged (39–51 years) males with white-collar jobs. The body mass indices of volunteers were 23.7–30.3 (mean ± SD: 26.6 ± 2.2). No volunteer was previously diagnosed with metabolic diseases or hypertension. This study was approved by the Institutional Review Board of Wonju Christian Hospital (IRB No. CR319063) and the study procedures were conducted in accordance with the Helsinki Declaration. All subjects provided written informed consent to participate in the study.

### 2.2. Hyperbaric Oxygen Therapy (HBOT)

**Figure 1:**
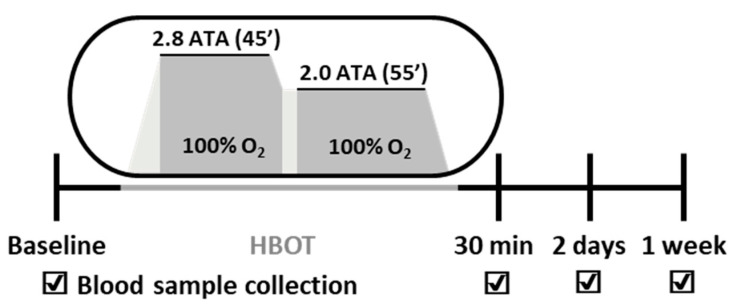
Experimental design of this pilot study. ATA, atmospheres absolute; HBOT, hyperbaric oxygen therapy.

HBOT was performed in a single or multiplace hyperbaric chamber (IBEX Medical Systems, Seoul, Korea). Compression was achieved to 2.8 atmospheres absolute (ATA) for 45 min and then 2.0 ATA for 55 min (Figure 1). For all participants, blood samples were collected before (baseline), immediately, 2 days, and 7 days after HBOT.

### 2.3. Blood Collection and Biochemical Analysis

Blood samples were collected at four different time points: baseline, immediately after a bout of HBO exposure, and after 2 days and 7 days of recovery, matching the time schedule of the trial. Samples were drawn from the antecubital vein and serum was separated, frozen, and stored at −80 °C until subsequent analysis. Biochemical measurements were performed using an automated clinical chemistry analyzer (Roche Cobas^®^ 8000, Roche, Mannheim, Germany) with the manufacturer’s reagents and calibrators at a certified central laboratory (SCL, Seoul, Korea). Triglyceride (TG), total cholesterol (TC), high density lipoprotein cholesterol (HDL-C), low density lipoprotein cholesterol (LDL-C), γ-glutamyltransferase (γ-GT), and uric acid levels were measured by enzymatic-colorimetric methods. Aspartate aminotransferase (AST), alanine aminotransferase (ALT), creatinine, albumin, blood urea nitrogen (BUN), and total protein were measured by colorimetric methods. Blood glucose was measured using the hexokinase method.

### 2.4. QMAP Assays

Quantamatrix Multiplexed Assay Platform (QMAP) analyses for the measurements of serum biomarkers were performed using shape-encoded magnetic microdisks and a QMAP 2.0 (Quantamatrix Inc., Seoul, Korea). Microdisks were hybridized with capture antibodies and reacted with samples and detection antibodies. Target-specific antibodies were purchased (R&D systems, Minneapolis, MN, USA) and product information (capture/detection antibody) is as follows: FGF21 (#DY2539/#BAF2539), GDF15 (#MAB957-100/#BAF940), adiponectin (#DY1065/#DY1065), leptin (#MAB398-100/#BAM398), retinol binding protein 4 (RBP4) (#MAB33781-100/#DY3378), and dickkopf-related protein 1 (DKK1) (#MAB10962-100/#BAF1096). After washing, microdisks were conjugated with streptavidin-R-phycoerythrin (Prozyme, Agilent Technologies, Santa Clara, CA, USA; #PJRS20). Fluorescence microscopic images were obtained using QMAP 2.0 and fluorescence intensities of all microdisks in the image were automatically analyzed using the software provided by QMAP.

### 2.5. ELISA Assays

Serum levels of biomarkers were validated by enzyme-linked immunosorbent assay (ELISA) using commercial kits targeting GDF15 (Quantikine, DGD150), FGF21 (Quantikine, DF2100), adiponectin (Quantikine, DRP300), leptin (Quantikine, DLP00), DKK1 (Quantikine, DKK100), and RBP4 (Quantikine, DRB400) according to the manufacturer’s instructions (R&D System, Minneapolis, MN, USA).

### 2.6. Statistical Analysis

Statistical analyses were conducted with GraphPad Prism 6.0 (GraphPad Software, Inc., San Diego, CA, USA). Data were assessed for normality of distribution using the Shapiro–Wilk test and expressed as the means ± standard deviations. A paired *t*-test or Wilcoxon signed-rank non-parametric test was used to compare between the baseline and last follow-up. Pearson’s correlation analysis was used for assessing the association between different serum biomarkers. To assess the effect of acute HBOT on serum biomarker levels at the four different time points, pairwise repeated measure single-factor analysis of variance (ANOVA) was performed with Greenhouse–Geisser correction and Tukey’s multiple comparison test or a Friedman test with Dunn’s multiple comparison correction test, as appropriate. All statistical tests were two-sided, and *p*-values less than 0.05 were considered statistically significant.

## 3. Results

There was an increase in the TG levels 1 week after HBOT, whereas other parameters, such as glucose and cholesterol, were not altered by hyperbaric oxygen. Neither liver function nor kidney function indicators were affected by HBOT intervention (Table 1).

**Figure 2:**
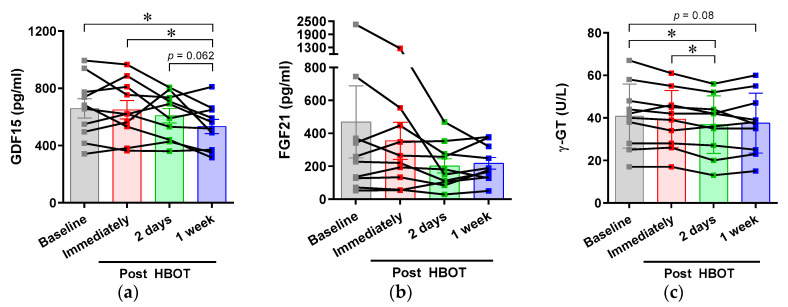
Effects of acute hyperbaric oxygen exposure on the serum levels of mitochondrial and oxidative stress biomarkers. Blood from volunteers was taken at different time points before and after HBOT. The serum levels of (**a**) growth/differentiation factor 15 (GDF15), (**b**) fibroblast growth factor 21 (FGF21), and (**c**) **γ**-glutamyltransferase (**γ**-GT) were measured using a Quantamatrix Multiplexed Assay Platform (QMAP) system. HBOT, Hyperbaric oxygen therapy. * *p* < 0.05.

To evaluate the effect of HBOT on metabolic stresses, we measured the serum levels of the following humoral factors related to cellular stress: GDF15, FGF21, and *γ*-GT. The expressions of GDF15 and FGF21 are increased upon mitochondrial stress, which elicits a protective response against pathogenic progression. *γ*-GT is involved in glutathione metabolism to preserve redox homeostasis [21]. Elevated serum γ-GT predominantly indicates liver disease, but is also detected in cardiovascular and metabolic diseases related to oxidative stress [22]. We observed that the serum GDF15 concentration was decreased at 1 week after HBOT. There was also a significant reduction of γ-GT at 2 days after HBOT. The circulating level of FGF21 tends to be reduced by HBOT, but this change was not significant (Figure 2).

**Figure 3:**
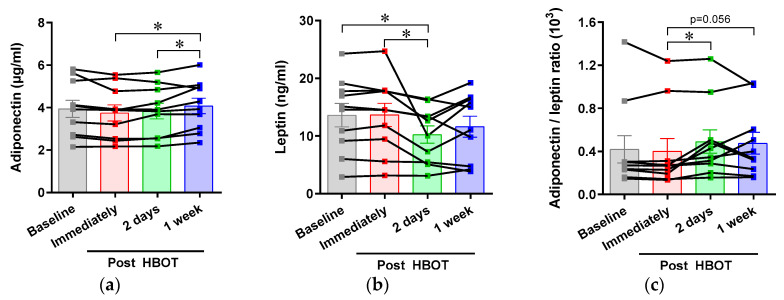
Effects of an acute hyperbaric oxygen exposure on the serum levels of adipokines. Blood from volunteers was taken at different time points before and after HBOT. Serum levels of (**a**) adiponectin and (**b**) leptin were measured using a QMAP system, and (**c**) adiponectin to leptin ratio was calculated from the individual’s adipokine levels. HBOT, Hyperbaric oxygen therapy. * *p* < 0.05.

**Figure A1:**
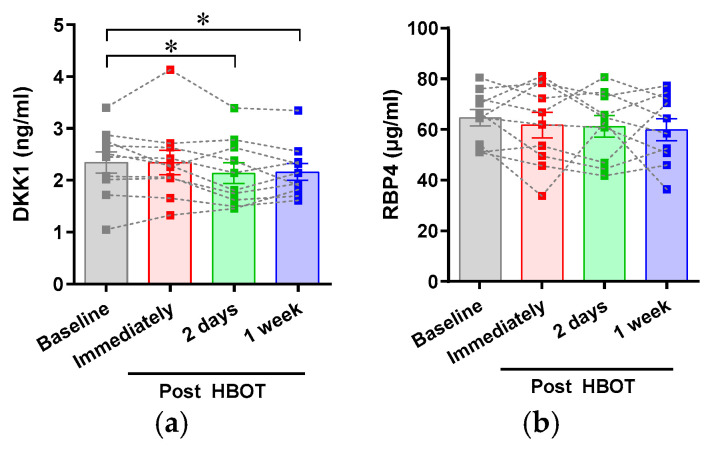
Effects of acute hyperbaric oxygen exposure on the serum levels of metabolism-related cytokines. Serum levels of DKK1 (**a**) and RBP4 (**b**) were measured using a QMAP system. DKK1, dickkopf-related protein 1; RBP4, retinol binding protein 4. * *p* < 0.05.

To estimate the influence of HBOT on whole body metabolism, we measured the serum concentrations of adipokines, adiponectin, and leptin. Upon metabolic stress, such as visceral obesity with systemic inflammation, there was a decrease in serum adiponectin and an increase in leptin [23]. We demonstrated that HBOT increased circulating adiponectin and reduced leptin levels, and, as a result, increased the adiponectin/leptin ratio (Figure 3). We also measured the levels of DKK1 and RBP4, as metabolism-related humoral factors; the levels of both these components are reported to be increased in obesity [24]. The circulating level of DKK1 was reduced by HBOT, but that of RBP4 was not affected (Figure A1).

## 4. Discussion

We performed a pilot intervention study of HBOT in volunteers with no known metabolic diseases. The HBOT program, consisting of 2.8 ATA for 45 min and 2.0 ATA for 55 min, showed a decrease in serum levels of mitochondrial and oxidative stress biomarkers within 2 days or 1 week after receiving HBOT. We also observed an increase in the adiponectin/leptin ratio, suggesting an amelioration of metabolic stress by one bout of HBOT. There were significant inter-correlations among serum levels of stress biomarkers and their changes by HBOT (Table A1 and Table A2). For instance, the HBOT-induced reduction in serum GDF15 level of study participants was closely correlated with those of FGF21 or γ-GT, which indicates the consistency of stress-relieving responses. This is the first study to assess the changes in the levels of metabolism-related serum biomarkers following HBOT. The metabolic benefit of HBOT could extend the therapeutic indication of hyperbaric oxygen to a wide range of chronic diseases.

The molecular mechanisms of HBOT to relieve metabolic stress were not investigated here. Instead, we infer that the primary functional consequences of hyperbaric oxygen could be related to mitochondria, which use most of the intracellular oxygen and produce ATP and water. There have been reports of the detrimental actions of hyperbaric oxygen, known as the Paul Bert effect, which include disturbance in the central nervous system and injuries to lungs and eyes [25]. In newborn babies, prolonged exposure to high oxygen pressure causes retrolental fibroplasia and hyperoxic myopia associated with oxidative stress [26]. However, a low range of oxidative stress induced by HBOT also has physiologic roles, such as promoting wound healing and improving tissue survival [25,27]. The reactive oxygen species production by hyperbaric oxygen also affects mitochondrial functions such as regulation of mitochondrial ion channels and energy metabolism [28]. An accumulation of experimental evidence has shown that HBOT protects against mitochondrial dysfunction [11] and improves mitochondrial biogenesis and respiration [14,29], thus supporting the beneficial and therapeutic effects of HBOT.

GDF15 is a member of the transforming growth factor β/bone morphogenetic protein superfamily, expressed in the kidney, liver, adipose tissue, lung, pancreas, heart, brain, and skeletal muscles [19,30]. Various stress and tissue injuries upregulate GDF15 expression and its release into circulation. Hypoxia and anoxia also increase GDF15 expression in retinal pigment epithelial cells, colon cancer, prostate cancer, and glioblastoma [30]. The physiologic action of GDF15 has not been clearly elucidated, but it reduces appetite and increases energy expenditure; thus, it relieves metabolic stress [31]. Consistently, GDF15 also modulates mitochondrial functions including biogenesis, thermogenesis, and fatty acid metabolism [19]. The γ-GT level also increases upon oxidative stress to augment the glutathione-mediated antioxidant defense mechanism [21]. An elevated serum γ-GT level is correlated with cardiometabolic risks in diabetes, hypertension, dyslipidemia, and obesity [21]. Therefore, reduced levels of GDF15 and γ-GT imply the attenuation of existing mitochondrial and oxidative stresses by HBOT, which suggest therapeutic potential for metabolic diseases or neurodegenerative diseases associated with oxidative stress and mitochondrial dysfunction.

**Figure A2:**
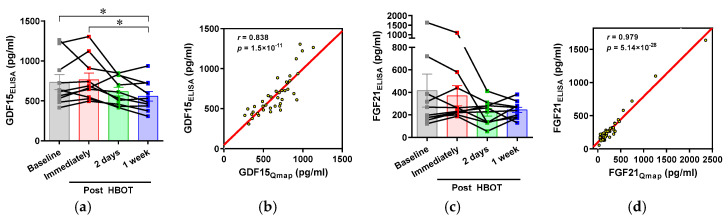
Validation of the QMAP analysis of serum growth differentiation factor 15 (GDF-15) and fibroblast growth factor 21 (FGF-21) concentrations using ELISA analysis throughout the pilot study following acute exposure to HBOT. Serum levels of GDF15 (**a**) and FGF21 (**c**) measured by ELISA, and their correlation (**b**, for GDF15; **d**, for FGF21) with the values determined by QMAP analysis. QMAP, Quantamatrix Multiplexed Assay Platform; *r*, Pearson correlation coefficient. Subscripts, _Qmap_ and _ELISA_, indicate the performed methods for quantifying serologic biomarker levels. * *p* < 0.05.

**Figure A3:**
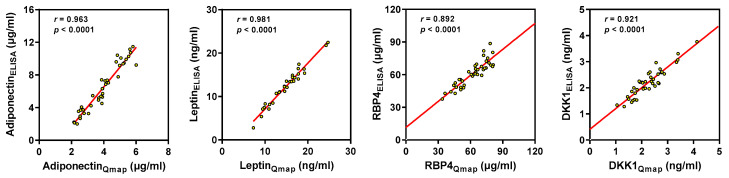
Validation of the QMAP analysis of serum adiponectin, leptin, retinal binding protein 4 (RBP4), and dickkopf-related protein 1 (DKK1) concentrations using ELISA analysis. QMAP, Quantamatrix Multiplexed Assay Platform; *r*, Pearson correlation coefficient. Subscripts, _Qmap_ and _ELISA_, indicate the performed methods for quantifying serologic biomarker levels.

In this study, we used a multiplexed QMAP system to measure the concentrations of biomarkers from volunteer’s serum samples. The accuracy of the QMAP measurement was validated via ELISA, and a good correlation was found between the two methods (Figure A2 and Figure A3). There are complexities and diversities in the disease progression of metabolic diseases and age-related diseases. Accordingly, the provision of diversified diagnosis and prognosis based on specific pathogenic mechanisms is useful for estimating the pathologic status and establishing therapeutic plans. Reliable results demonstrate the effectiveness of multilayered biomarker measurements by QMAP analyses, which could fulfil the unmet need for multidimensional diagnostic approaches to assess complicated pathogenesis.

We observed changes in the levels of stress biomarkers and metabolic humoral factors in response to HBOT, implying the improvement of metabolic conditions related to intracellular organellar functions and redox homeostasis. However, this pilot intervention test with a small number of participants is limited as a basis from which to draw conclusions regarding the beneficial effect of HBOT. Further large-scaled interventional studies with control and disease groups could elucidate the molecular mechanisms underlying HBOT’s beneficial effect and provide novel treatment strategies for metabolic dysfunction, in addition to the conventional therapeutic indications of HBOT.

## 5. Conclusions

Single hyperbaric oxygen exposure was shown to attenuate mitochondrial stress and oxidative stress with the improvement of metabolic status in this pilot intervention study. We suggest that HBOT shows novel therapeutic potential to alleviate metabolic morbidities precipitated by oxidative stress and mitochondrial dysfunction.

## Figures and Tables

**Table 1:** Serum biochemical variables at baseline and following acute exposure to hyperbaric oxygen therapy (HBOT).

Variables	Baseline	1 Week Post HBOT	*p*-Value
Glucose (mg/dL)	104.4 ± 19.1	105.0 ± 35.4	0.967
TC (mg/dL)	186.5 ± 17.8	185.8 ± 24.8	0.921
TG (mg/dL) *	154.9 ± 58.4	190.4 ± 66.1	0.012
HDL-C (mg/dL)	47.2 ± 7.5	45.2 ± 7.2	0.206
LDL-C (mg/dL)	121.5 ± 23.1	116.1 ± 23.8	0.272
ALT (U/L) *	39.6 ± 21.2	36.1 ± 12.2	0.483
AST (U/L) *	29.4 ± 5.5	29.7 ± 5.3	0.812
Total protein (g/dL)	7.45 ± 0.26	7.30 ± 0.29	0.143
Albumin (g/dL) *	4.64 ± 0.25	4.58 ± 0.23	0.301
BUN (mg/dL)	12.74 ± 1.65	12.43 ± 1.76	0.599
Creatinine (mg/dL)	0.82 ± 0.05	0.81 ± 0.04	0.532
Uric acid (mg/dL)	6.37 ± 0.85	6.11 ± 0.95	0.106

ALT, alanine aminotransferase; AST, aspartate aminotransferase; BUN, blood urea nitrogen; LDL-C, low density lipoprotein cholesterol; HDL-C, high density lipoprotein cholesterol; TC, total cholesterol; TG, triglyceride. Statistical analyses were performed using Student’s paired *t*-test or * Wilcoxon matched-pairs signed-rank test. Values represent the means ± SD.

## Appendix A

**Table A1:** Pearson’s correlation matrix among serum biomarker levels.

	GDF15	FGF21	γ-GT	Adiponectin	Leptin	A/L	Triglyceride
**GDF15**	1						
**FGF21**	0.214	1					
**γ-GT**	−0.023	0.453 **	1				
**Adiponectin**	0.065	0.072	0.305	1			
**Leptin**	0.487 **	0.115	0.325 *	0.075	1		
**A/L**	−0.356 *	−0.138	−0.380 *	0.323 *	−0.801 **	1	
**Triglyceride**	−0.386 *	0.018	0.163	−0.23	−0.072	−0.07	1

Values are Pearson correlation coefficients (r); * *p* < 0.05, ** *p* < 0.01. GDF15, growth differentiation factor 15; FGF21, fibroblast growth factor 21; γ-GT, γ-glutamyltransferase; A/L, adiponectin to leptin ratio.

**Table A2:** Pearson’s correlation matrix for changes in biomarker levels from baseline to 1 week after HBOT.

	ΔGDF15	ΔFGF21	Δγ-GT	ΔAdiponectin	ΔLeptin	ΔA/L	ΔTriglyceride
**ΔGDF15**	1						
**ΔFGF21**	0.673 **	1					
**Δγ-GT**	0.539 *	0.386	1				
**ΔAdiponectin**	−0.01	−0.504 *	0.17	1			
**ΔLeptin**	0.557 **	0.317	0.737 ***	0.17	1		
**ΔA/L**	−0.428	−0.326	−0.289	0.088	−0.612 **	1	
**ΔTriglyceride**	0.244	−0.053	−0.309	0.031	−0.069	−0.423	1

Values are Pearson correlation coefficients (*r*); * *p* < 0.1, ** *p* < 0.05, *** *p* < 0.01. Δ, 1 week after – before HBOT; GDF15, growth differentiation factor 15; FGF21, fibroblast growth factor 21; γ-GT, γ-glutamyltransferase; A/L, adiponectin to leptin ratio.
